# Routinely Available Prognostic Factors for Adverse Outcomes in Adults with Complicated Parapneumonic Effusion and Empyema: A Systematic Review and Meta-Analysis

**DOI:** 10.3390/jcm15207778

**Published:** 2026-10-09

**Authors:** Madalina Alexandra Balica, Catalin Balan, Delia Hutanu, Norbert Wellmann, Lavinia Balan, Norberth-Istvan Varga, Iulia Cristina Bagiu, Ovidiu Rosca, Cristian Ion Mot, Corneluta Fira-Mladinescu, Ovidiu Fira-Mladinescu

**Affiliations:** 1 Doctoral School, “Victor Babes” University of Medicine and Pharmacy, Eftimie Murgu Square 2, 300041 Timisoara, Romania; madalina.balica@umft.ro (M.A.B.); bcatalin43@yahoo.com (C.B.); 2 Department of Biology, Chemistry-Biology-Geography Faculty, West University of Timisoara, Pestalozzi Street, No 16 A, 300115 Timisoara, Romania; delia.hutanu@e-uvt.ro; 3 Pulmonology University Clinic, Clinical Hospital of Infectious Diseases and Pneumophthisiology Dr. Victor Babeș, 300310 Timisoara, Romania; norbert.wellmann@umft.ro; 4 Center of Research and Innovation in Personalized Medicine of Respiratory Diseases (CRIPMRD), “Victor Babes” University of Medicine and Pharmacy, 2 Eftimie Murgu Square, 300041 Timisoara, Romania; mladinescu@umft.ro; 5 Department of Obstetrics and Gynecology, “Victor Babes” University of Medicine and Pharmacy, Eftimie Murgu Square, No. 2, 300041 Timisoara, Romania; lavinia.balan@umft.ro; 6 Department of Nursing, “Victor Babes” University of Medicine and Pharmacy, Eftimie Murgu Square, No. 2, 300041 Timisoara, Romania; norberth.varga@umft.ro; 7 Multidisciplinary Research Center on Antimicrobial Resistance (MULTI-REZ), Microbiology Department, “Victor Babes” University of Medicine and Pharmacy, Eftimie Murgu Square No. 2, 300041 Timisoara, Romania; bagiu.iulia@umft.ro; 8 Department of Microbiology, “Victor Babes” University of Medicine and Pharmacy, Eftimie Murgu Square No. 2, 300041 Timisoara, Romania; 9 Department XIII, Discipline of Infectious Diseases, “Victor Babeș” University of Medicine and Pharmacy, Eftimie Murgu Square 2, 300041 Timisoara, Romania; ovidiu.rosca@umft.ro; 10 Department of Ear Nose Throat, “Victor Babes” University of Medicine and Pharmacy, Eftimie Murgu Square, No. 2, 300041 Timisoara, Romania; 11 Discipline of Hygiene, Department of Microbiology, Center for Studies in Preventive Medicine, “Victor Babes” University of Medicine and Pharmacy, 300041 Timisoara, Romania; 12 Second Department of Pulmonology, Clinical Hospital of Infectious Diseases and Pulmonology “Victor Babes” Timisoara, Gheorghe Adam Street No. 13, 300310 Timisoara, Romania

**Keywords:** pleural infection, pleural empyema, complicated parapneumonic effusion, prognostic factors, adverse outcomes, mortality, RAPID score, systematic review, meta-analysis

## Abstract

**Background**: Pleural infection remains associated with substantial morbidity, mortality, and frequent treatment escalation. This systematic review and meta-analysis evaluated routinely available prognostic factors associated with adverse clinical outcomes in adults with pleural infection, including complicated parapneumonic effusion and pleural empyema. **Methods**: PubMed, Scopus, Web of Science Core Collection, and the Cochrane Library were searched from inception to 6 July 2026. Studies evaluating routinely available clinical, biochemical, microbiological, pleural fluid, radiological, or composite prognostic factors were included. Methodological quality was assessed using the Newcastle–Ottawa Scale (NOS). Comparable data were synthesized using random-effects meta-analysis. **Results**: Thirty studies involving 8443 participants were included. The evaluated outcomes comprised treatment failure, short-term mortality, need for surgery, intensive care unit admission, recurrence, readmission, and length of hospital stay. Five studies (877 participants) contributed to the RAPID score meta-analysis. High-risk RAPID classification was associated with greater short-term mortality than combined low- and intermediate-risk classification (RR 4.52, 95% CI 2.71–7.55; primary OR 6.09, 95% CI 3.25–11.38; I^2^ = 0% for the OR analysis). In a limited meta-analysis of three studies (1239 participants), each 10-year increase in age was associated with higher odds of short-term mortality (OR 1.54, 95% CI 1.02–2.30; I^2^ = 30.4%); however, mortality was assessed in hospital in two studies and at 90 days in one study. Additional prognostic factors associated with adverse outcomes included comorbidity burden, renal dysfunction, hypoalbuminemia, serial C-reactive protein trajectories, bacteremia, pleural fluid biochemical markers, pleural thickening, loculation, and delayed or ineffective drainage, although heterogeneous reporting precluded further quantitative synthesis. **Conclusions**: The RAPID score is the most consistently supported routinely available predictor of short-term mortality in adults with pleural infection. Increasing age may also be prognostic, but its pooled estimate is based on only three studies with different mortality windows and should be interpreted cautiously. Future prospective multicenter studies using standardized outcome definitions are needed to validate additional prognostic markers and improve risk-guided clinical decision-making.

## 1. Introduction

Pleural infection, encompassing complicated parapneumonic effusion (CPE) and pleural empyema, remains a severe infection of the pleural space and a major global healthcare burden. Despite advances in antimicrobial therapy, image-guided drainage, intrapleural enzyme therapy, and minimally invasive thoracic surgery, it continues to cause substantial morbidity, prolonged hospitalization, frequent invasive procedures, and high mortality [1,2,3,4].

Pleural infection most commonly develops as a complication of bacterial pneumonia. Parapneumonic pleural effusion occurs in approximately 20–57% of hospitalized patients with pneumonia, and 5–10% progress to pleural infection requiring drainage or surgery [1,2]. Importantly, nearly one-third of patients present without radiological evidence of pneumonia, illustrating the heterogeneity of its clinical presentation [1]. Its incidence has also continued to rise worldwide, with population-based studies from Europe and North America reporting increasing hospital admissions over the past two decades; in England, the incidence of pleural empyema increased from 6.44 per 100,000 hospital admissions in 2008 to 8.38 per 100,000 hospital admissions in 2017 [5]. Population ageing, chronic comorbidities, immunosuppression, antimicrobial resistance, changing microbiological patterns, and improved diagnostic imaging may all contribute to this trend [1,2,4]. Outcomes remain poor: mortality approaches 10% at 30 days and 20–35% at one year, while repeated pleural interventions, prolonged hospitalization, intensive care admission, and impaired quality of life further increase the burden of disease [1,2,3,4].

Treatment is based on prompt antimicrobial therapy and effective drainage of the infected pleural collection. Image-guided chest tube drainage remains the recommended first-line intervention, while combined intrapleural tissue plasminogen activator (tPA) and deoxyribonuclease (DNase) has become an established treatment option following the landmark MIST-2 trial [3,6]. Yet, despite guideline-based management, a substantial proportion of patients experience treatment failure, persistent pleural sepsis, prolonged hospitalization, or death [1,2,3]. Identifying these patients early could allow timely escalation to intrapleural enzyme therapy or surgery before advanced pleural organization and persistent sepsis develop [1,3,6]. Accurately recognizing high-risk patients at presentation, however, remains a major challenge in contemporary pleural medicine [1,2,3,7].

This has prompted growing interest in routinely available prognostic factors that could support early risk stratification and individualized treatment [2,7,8,9]. Clinical, laboratory, microbiological, and pleural fluid variables have an important practical advantage over emerging molecular biomarkers or artificial intelligence-based models: they are inexpensive, immediately available, and routinely measured at the bedside [2,7,9]. Observational studies have investigated demographic characteristics, comorbidities, inflammatory biomarkers, renal function, microbiological findings, and pleural fluid biochemistry, but their reported prognostic value has been inconsistent [1,2,7,8,9,10]. The RAPID score is currently the best-validated prognostic model and is recommended by international guidelines for mortality risk stratification [1,3,8,10]. However, it was developed primarily to predict short-term mortality and incorporates only a limited number of routinely available variables, potentially overlooking other prognostic information identified across observational studies [7,8,10].

Despite the rapid expansion of prognostic research in pleural infection, the evidence remains fragmented [2,7,8,9]. Studies vary substantially in patient populations, predictor definitions, treatment strategies, outcomes, and statistical methods, and even treatment failure lacks a universally accepted definition [1,2,3,7]. These differences make direct comparisons difficult and limit quantitative synthesis for many routinely available prognostic factors. Previous reviews have focused mainly on diagnosis, management, therapeutic interventions, or individual prognostic models—particularly the RAPID score—rather than the full spectrum of routinely available prognostic factors [2,6,8,9,10]. To the best of our knowledge, no previous systematic review and meta-analysis has comprehensively synthesized evidence on routinely available clinical, biochemical, microbiological, and pleural fluid factors associated with the broader range of adverse outcomes, including treatment failure, mortality, need for surgery, intensive care unit admission, recurrence, and prolonged hospitalization. A clearer synthesis of these predictors could improve early bedside risk stratification and support more timely, individualized treatment decisions.

Therefore, this systematic review and meta-analysis aimed to identify, critically appraise, and synthesize evidence on routinely available prognostic factors associated with adverse clinical outcomes in adults with complicated parapneumonic effusion and pleural empyema. Outcomes of interest were treatment failure, mortality, need for surgery, intensive care unit admission, hospital readmission, recurrence, and prolonged hospital stay. Where sufficiently comparable data were available, quantitative synthesis was performed to identify the prognostic factors most consistently associated with adverse clinical outcomes.

## 2. Materials and Methods

### 2.1. Search Strategy

This systematic review and meta-analysis was conducted and reported in accordance with the Preferred Reporting Items for Systematic Reviews and Meta-Analyses (PRISMA) 2020 statement [11]. As Supplementary Material you can find the PRISMA checklist. The review protocol was prospectively registered in the International Prospective Register of Systematic Reviews (PROSPERO; registration number: CRD420261448850).

A comprehensive literature search was performed in PubMed, Scopus, Web of Science, and the Cochrane Library from database inception to 6 July 2026. The search strategy was developed according to a predefined PECO (Population, Exposure, Comparator, Outcome) framework and combined controlled vocabulary (Medical Subject Headings [MeSH], where applicable) with free-text terms related to pleural infection, complicated parapneumonic effusion, pleural empyema, prognosis, prognostic factors, treatment failure, drainage failure, mortality, and surgical intervention. Database-specific search strategies were adapted according to the indexing system and search syntax of each database. The complete search strategies for all databases are provided in Supplementary File S1.

The search was restricted to studies involving adult human participants and published in English. In addition, the reference lists of all included studies and relevant review articles were manually screened to identify potentially eligible studies that were not retrieved through the electronic database search.

### 2.2. Review Question and PECO Framework

The review aimed to identify and synthesize the available evidence regarding routinely available prognostic factors associated with adverse clinical outcomes in adults with complicated parapneumonic effusion and pleural empyema. The review question was formulated according to the PECO (Population, Exposure, Comparator, Outcome) framework. The population comprised adults with complicated parapneumonic effusion, pleural empyema, or pleural infection. The exposure included routinely available clinical, biochemical, microbiological, and pleural fluid prognostic factors. Comparators consisted of patients without the investigated prognostic factor or with different levels of the same prognostic variable, where applicable. The outcomes of interest included treatment failure, mortality, need for surgical intervention, intensive care unit admission, hospital readmission, recurrence, and prolonged length of hospital stay.

### 2.3. Study Selection

All records identified through the database searches were exported and imported into Rayyan web (Rayyan Systems Inc., Cambridge, MA, USA) for study management, duplicate removal, and screening. After duplicate records were removed, two reviewers independently screened the titles and abstracts of all retrieved studies according to the predefined eligibility criteria. Potentially eligible studies underwent full-text review to determine final eligibility.

**Figure 1:**
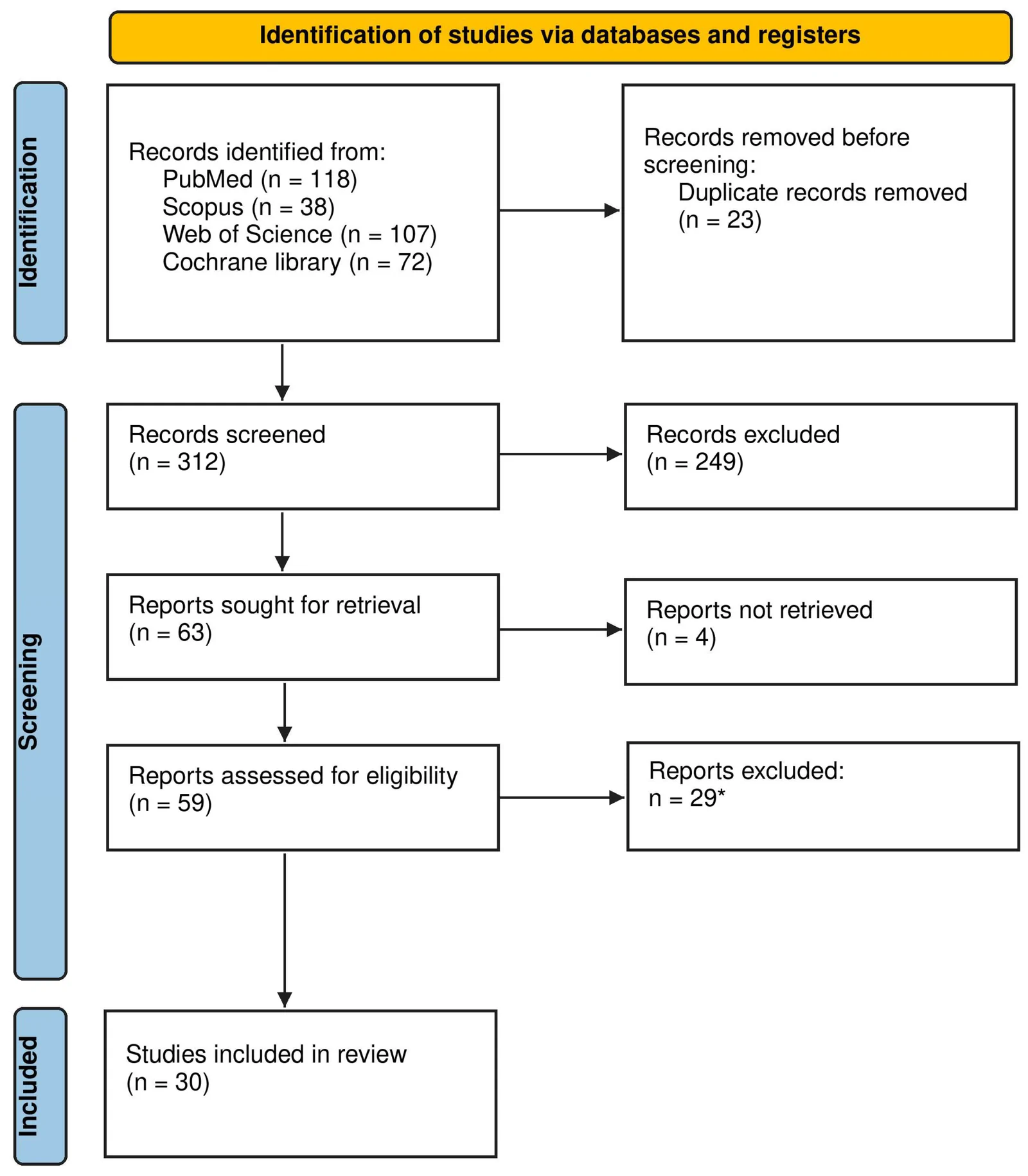
Study selection process according to PRISMA guidelines. * Reasons for exclusion are presented in Supplementary File S2, Table S1.

Any disagreements regarding study selection were resolved through discussion and consensus. When consensus could not be reached, a third reviewer was consulted to reach the final decision. Reasons for exclusion at the full-text stage were documented. The study selection process was documented using a PRISMA flow diagram, shown in Figure 1.

### 2.4. Eligibility Criteria

Studies were considered eligible if they fulfilled the predefined PECO criteria. Eligible studies included adult patients (≥18 years) with complicated parapneumonic effusion, pleural empyema, or pleural infection. Studies were required to evaluate the association between at least one routinely available prognostic factor and one or more predefined adverse clinical outcomes. Eligible prognostic factors included routinely available clinical, biochemical, microbiological, pleural fluid, or prognostic score variables, such as demographic characteristics, comorbidities, inflammatory biomarkers, pleural fluid biochemical parameters, microbiological findings, or validated prognostic scores (e.g., RAPID score).

Eligible studies were required to report at least one clinically relevant outcome, including treatment failure, failure of chest tube drainage, need for treatment escalation, surgical intervention (including video-assisted thoracoscopic surgery [VATS] or open decortication), all-cause mortality, in-hospital mortality, 30-day mortality, 90-day mortality, intensive care unit (ICU) admission, hospital readmission, or length of hospital stay. As no universally accepted definition of treatment failure exists, the definition reported by each individual study was accepted.

Eligible study designs included prospective and retrospective cohort studies, case-control studies, registry-based studies, and secondary analyses of clinical trials, provided that original prognostic data were reported.

Studies were excluded if they involved pediatric populations (unless adult data were reported separately), tuberculous pleural disease, malignant pleural effusions without pleural infection, postoperative or trauma-related pleural infections without separate reporting of parapneumonic empyema, or animal studies. Case reports, small case series without prognostic analyses, conference abstracts without sufficient extractable data, editorials, letters, narrative reviews, systematic reviews, meta-analyses, clinical practice guidelines, study protocols, and trial registrations without published results were also excluded. In addition, studies focusing exclusively on therapeutic interventions, diagnostic accuracy, or descriptive microbiology were excluded unless they evaluated the association between at least one routinely available prognostic factor and a relevant clinical outcome. Studies that did not report an association between a routinely available prognostic factor and treatment failure or another predefined adverse clinical outcome were also excluded.

### 2.5. Data Extraction

Data extraction was performed independently by two reviewers using a standardized data extraction form developed specifically for this systematic review. The extracted data included the following study characteristics: first author, year of publication, country, study design, study setting, sample size, patient demographics, and follow-up duration.

When reported, the following prognostic factors were extracted: routinely available clinical characteristics, comorbidities, laboratory biomarkers, microbiological findings, pleural fluid parameters, and validated prognostic scores. In addition, data on the assessed clinical outcomes were collected, including treatment failure, failure of chest tube drainage, need for treatment escalation, surgical intervention (including VATSor open decortication), mortality, intensive care unit admission, hospital readmission, recurrence, and length of hospital stay.

Where available, effect estimates (e.g., odds ratios, hazard ratios, or risk ratios), corresponding 95% confidence intervals, *p*-values, and variables included in multivariable analyses were extracted. For studies considered eligible for quantitative synthesis, additional data required for the meta-analysis, including the number of participants, number of events, and summary effect estimates, were extracted whenever available. When multiple publications reported data from the same study population, the publication providing the most complete and relevant data was included to avoid duplication.

Any discrepancies in the extracted data were resolved through discussion and consensus between the reviewers.

### 2.6. Quality Assessment

The methodological quality of the included observational studies was independently assessed by two reviewers using the Newcastle–Ottawa Scale (NOS) [12]. The NOS evaluates three domains: Selection, Comparability, and Outcome, with a maximum score of nine stars. Studies scoring 7–9 stars were considered high quality, those scoring 5–6 stars were considered moderate quality, and those scoring fewer than 5 stars were considered low quality. Disagreements between reviewers were resolved through discussion and consensus. Detailed study-level assessments are presented in Supplementary File S2, Table S2.

### 2.7. Statistical Analysis

A qualitative narrative synthesis was performed for all included studies. Meta-analysis was undertaken when at least three studies evaluated the same prognostic factor using sufficiently comparable predictor definitions, outcome measures, follow-up periods, and effect estimates.

Statistical analyses were performed using R software (version 4.4.1; R Foundation for Statistical Computing, Vienna, Austria) with the meta and metafor packages [13]. Pooled effect estimates were calculated using a random-effects model with the restricted maximum likelihood (REML) estimator. Because only a small number of studies were available for each quantitative synthesis, 95% confidence intervals (CIs) were calculated using the modified Hartung–Knapp adjustment. Odds ratios (ORs), risk ratios (RRs), and hazard ratios (HRs) were synthesized separately and were not combined within the same meta-analysis. When multiple adjusted models were reported, estimates from the most fully adjusted model were preferentially extracted. If adjusted estimates were unavailable, unadjusted ORs were calculated from study-level event counts whenever sufficient data were available.

For continuous prognostic factors, effect estimates were harmonized to clinically interpretable units before pooling. Specifically, age estimates reported per 1-year increase were converted to represent a 10-year increase. For the RAPID score analysis, the high-risk category (score 5–7) was compared with the combined low- and intermediate-risk categories (score 0–4), using short-term mortality (3-month or 90-day mortality) as the primary outcome.

Statistical heterogeneity was assessed using Cochran’s Q test and quantified using the I^2^ statistic and between-study variance (τ^2^) [14]. Sensitivity analyses included leave-one-out analyses and comparison with fixed-effect models. We planned to assess publication bias using funnel plots and Egger’s regression test when at least 10 studies were available for a given meta-analysis. A two-sided *p* value < 0.05 was considered statistically significant.

## 3. Results

### 3.1. Overview of Selected Studies

The systematic literature search identified 335 records across four electronic databases (PubMed, Scopus, Web of Science, and the Cochrane Library). After duplicate removal, the remaining records underwent title and abstract screening, resulting in 63 studies selected for full-text assessment. Four reports could not be retrieved. Following full-text review, 30 studies met the predefined eligibility criteria and were included in the qualitative synthesis. Studies reporting sufficiently comparable quantitative data were subsequently included in the meta-analysis [8,15,16,17,18,19,20,21,22,23,24,25,26,27,28,29,30,31,32,33,34,35,36,37,38,39,40,41,42,43]. Twenty-nine full-text articles were excluded for reasons including the absence of prognostic factor analyses, non-target populations, lack of relevant outcomes, non-original study designs, or insufficient extractable data [44,45,46,47,48,49,50,51,52,53,54,55,56,57,58,59,60,61,62,63,64,65,66,67,68,69,70,71,72] (Supplementary File S2, Table S1). The study selection process is presented in the PRISMA 2020 flow diagram (Figure 1).

The included studies were published between 1999 and 2026 and provide a comprehensive overview of routinely available prognostic factors associated with adverse clinical outcomes in adults with complicated parapneumonic effusion, pleural empyema, or pleural infection. Most studies were retrospective observational cohort studies, while the remainder comprised prospective cohort studies, including one randomized clinical trial with a secondary prognostic analysis, one prospective validation cohort, one database-based longitudinal cohort, and one multicenter retrospective cohort incorporating machine-learning prediction models [8,15,16,17,18,19,20,21,22,23,24,25,26,27,28,29,30,31,32,33,34,35,36,37,38,39,40,41,42,43].

Across the included reports, the summed sample size was 8443 adult participants, with individual study populations ranging from 34 to 1000 participants, reflecting considerable heterogeneity in study populations, healthcare settings, and study designs [8,14,15,16,17,18,19,20,21,22,23,24,25,26,27,28,29,30,31,32,33,34,35,36,37,38,39,40,41,42,43].

Across the included studies, investigators evaluated a broad range of routinely available prognostic factors, including demographic characteristics, comorbidities, inflammatory and biochemical biomarkers, microbiological findings, pleural fluid biochemical parameters, radiological features, and validated prognostic scores. The RAPID score was the most frequently investigated prognostic model, while other commonly evaluated predictors included age, diabetes mellitus, chronic kidney disease, malignancy, Charlson Comorbidity Index, serum albumin, urea, C-reactive protein, pleural fluid pH, pleural glucose, pleural lactate dehydrogenase, bacteremia, microbiological culture positivity, timing of pleural drainage or surgery, and radiological characteristics [8,15,16,17,18,19,20,21,22,23,24,25,26,27,28,29,30,31,32,33,34,35,36,37,38,39,40,41,42,43].

The reported outcomes were heterogeneous and included treatment failure, failure of chest tube drainage, need for treatment escalation, surgical intervention (including video-assisted thoracoscopic surgery and open decortication), mortality (in-hospital, 30-day, 90-day, and longer-term mortality), intensive care unit admission, recurrence, postoperative complications, hospital readmission, and length of hospital stay [8,15,16,17,18,19,20,21,22,23,24,25,26,27,28,29,30,31,32,33,34,35,36,37,38,39,40,41,42,43]. The principal characteristics of the included studies are summarized in Table 1.

### 3.2. Methodological Quality Assessment

The methodological quality of the included studies was assessed using the Newcastle–Ottawa Scale (NOS) for cohort studies. Overall, the included studies demonstrated moderate to high methodological quality, with most studies demonstrating moderate to high methodological quality (6–9 stars), with more than half achieving 8–9 stars. The highest scores were consistently observed in the Selection and Outcome domains, reflecting well-defined study populations, appropriate ascertainment of prognostic factors, and adequate outcome assessment.

Most studies enrolled representative cohorts of adults with pleural infection or pleural empyema and clearly reported their eligibility criteria. Clinical outcomes, including mortality, treatment failure, recurrence, and the need for surgical intervention, were primarily assessed using hospital records or institutional databases, and follow-up was generally sufficient to capture the outcomes of interest.

The principal methodological limitations were observed within the Comparability domain. Although many studies performed multivariable analyses to adjust for potential confounding factors, the variables included in these models differed considerably across studies. In addition, heterogeneity in patient populations, definitions of treatment failure, management strategies, and follow-up duration contributed to methodological variability. Most included studies were retrospective single-center cohorts, which may have introduced selection bias and limited the generalizability of the findings.

Overall, the methodological quality assessment indicates that the available evidence is supported by studies of predominantly moderate to high methodological quality, although the observational design and methodological heterogeneity of the included studies should be considered when interpreting the findings. Detailed Newcastle–Ottawa Scale assessments for each included study are provided in Supplementary File S2, Table S2.

### 3.3. Qualitative Synthesis of Prognostic Factors

#### 3.3.1. Prognostic Score

Among the prognostic models evaluated, the RAPID score was the most extensively investigated. Incorporating Renal function (urea), Age, Purulence, Infection source, and Dietary factors (serum albumin), it was originally developed to predict short-term mortality and subsequently evaluated across diverse clinical settings and populations included in this review [8,17,22,25,36].

Higher RAPID scores were consistently associated with increased short-term mortality and other adverse outcomes, including treatment failure, prolonged hospitalization, and treatment escalation [8,17,25,36]. High-risk patients had poorer survival than low- and intermediate-risk groups. The prospective multicenter PILOT validation study showed progressively increasing mortality across predefined RAPID risk categories, confirming the score’s reproducibility in an independent cohort [8].

Several studies also assessed the relationship between RAPID score and surgical management. Simon et al. found significantly lower survival in the high-risk category, with no significant difference between low- and intermediate-risk groups. Surgically treated patients had numerically higher survival than medically managed patients, but the difference was not statistically significant after stratification by RAPID risk category [21]. Similarly, Akboga et al. reported shorter survival with high RAPID scores and suggested that associations between RAPID score and surgical outcomes may partly reflect overlap between score components and clinical factors guiding surgical decisions [22].

Beyond mortality, higher RAPID scores were associated with longer hospital stays, greater healthcare resource utilization, and a higher likelihood of invasive management [25,36]. However, treatment decisions were also influenced by radiological findings, microbiological characteristics, comorbidities, and overall clinical status [8,21,22].

#### 3.3.2. Demographic and Clinical Predictors

Demographic and clinical factors were frequently evaluated. Advanced age was consistently associated with increased mortality, treatment failure, and poorer prognosis, and several studies identified it as an independent predictor [15,18,24,27,34,42]. However, in one surgical cohort, age was not independently associated with postoperative mortality after adjustment for comorbidities and sepsis-related complications [42].

Diabetes mellitus was also frequently investigated, but its independent prognostic value was inconsistent: some studies identified it as an independent predictor of adverse outcomes, whereas others found its association attenuated after adjustment for additional clinical variables [18,23,26,37,42].

Chronic kidney disease, malignancy, cardiovascular and chronic respiratory disease, and immunosuppression were also assessed. Chronic kidney disease and impaired renal function were consistently associated with poorer outcomes and higher mortality [17,24,27,42]. Malignancy and immunosuppression were associated with adverse outcomes in several cohorts, although effect sizes varied across populations and settings [27,40,42].

Greater overall comorbidity burden, particularly higher Charlson Comorbidity Index (CCI) values, was associated with increased mortality and treatment failure [34,41].

Markers of disease severity at presentation—including sepsis, respiratory failure, delayed presentation, and poor baseline functional status—were also associated with adverse outcomes. Systemic inflammatory response, organ dysfunction, or advanced pleural infection increased the likelihood of treatment escalation, surgery, prolonged hospitalization, or mortality [17,31,34,42].

Overall, advanced age, greater comorbidity burden, chronic kidney disease, malignancy, and severe systemic illness were the most frequently reported demographic and clinical predictors of adverse outcomes, although association strength varied across studies.

#### 3.3.3. Laboratory Biomarkers

Laboratory biomarkers were among the most extensively investigated prognostic factors across the included studies. Routine blood parameters reflecting systemic inflammation, nutritional status, renal function, and hematological abnormalities were frequently evaluated for their association with treatment failure, mortality, and other adverse clinical outcomes.

Among the investigated biomarkers, serum albumin was one of the most consistently reported prognostic factors. Lower serum albumin concentrations were associated with increased mortality, treatment failure, prolonged hospitalization, and higher RAPID scores across multiple studies [17,22,24,27,35,42]. Several studies also identified hypoalbuminemia as an independent predictor of adverse clinical outcomes in multivariable analyses [24,27,35,42].

Markers of renal dysfunction, particularly serum urea, were also consistently associated with unfavorable outcomes. Elevated serum urea concentrations were linked to increased mortality and greater disease severity in several cohorts [8,17,24,27]. Serum urea is also incorporated into the RAPID score as one of its five components.

C-reactive protein (CRP) was evaluated in multiple studies with variable results. Although elevated CRP concentrations generally reflected greater disease severity, their independent prognostic value was less consistent than that observed for serum albumin or urea. However, persistently elevated or slowly declining CRP levels during treatment were associated with poorer clinical outcomes and increased mortality in several cohorts [24,31,35].

Several studies additionally investigated hematological parameters, including hemoglobin concentration, total leukocyte count, neutrophil percentage, neutrophil-to-lymphocyte ratio (NLR), platelet count, and other routinely available blood indices. Lower hemoglobin concentrations and markers of an exaggerated inflammatory response were associated with prolonged hospitalization, treatment failure, and mortality in selected cohorts, although these findings were not consistently replicated across all studies [18,34,43].

#### 3.3.4. Pleural Fluid Biomarkers

Pleural fluid biomarkers were frequently assessed as predictors of treatment failure, mortality, recurrence, and surgical intervention, particularly pH, glucose, LDH, protein, and purulence, with variable prognostic value.

Low pleural glucose was associated with more advanced infection and poorer outcomes. A glucose level ≤ 40 mg/dL was more frequent in patients with recurrent bacterial empyema [26], although it was not consistently an independent predictor after adjustment [17,19].

Lower pleural pH generally reflected more advanced infection but showed inconsistent associations with adverse outcomes [17,19]. In surgical cohorts, it was assessed alongside age, comorbidities, pleural protein, and surgery timing as a predictor of mortality or treatment failure.

Pleural LDH showed more consistent prognostic value. Higher LDH independently predicted conversion from VATS to open thoracotomy, with a reported threshold >1854 IU/L [19], and was also an important predictor of intrapleural enzyme therapy failure in machine-learning models [31].

Absence of frank purulence was associated with successful medical treatment, while other baseline pleural fluid variables had limited independent value for treatment failure [15]. Purulence is also a RAPID score component. Pleural protein was less frequently studied and showed inconsistent associations with mortality and treatment failure [19].

Overall, elevated pleural LDH and low glucose showed the most consistent associations with treatment failure, recurrence, and surgical intervention, whereas pH, protein, and purulence had more variable prognostic value [15,17,19,26,31].

#### 3.3.5. Microbiological Predictors

Microbiological factors, including pleural fluid culture positivity, bacteremia, pathogen type, polymicrobial infection, antimicrobial resistance, and healthcare- versus community-acquired infection, were frequently evaluated, with variable prognostic value across studies and settings.

Positive pleural fluid cultures were generally associated with greater disease severity, prolonged hospitalization, treatment complexity, and failure, but were not consistently independent predictors of mortality after adjustment [18,30,35].

Bacteremia showed one of the strongest associations with adverse outcomes, including higher mortality, more severe systemic illness, and greater need for intensive care or surgery [24,34,41].

Outcomes also varied by pathogen type. Polymicrobial infections, Gram-negative organisms, and healthcare-associated pathogens were generally associated with poorer outcomes than typical community-acquired pathogens, although the impact of individual microorganisms varied with local epidemiology and resistance patterns [26,34,40].

Healthcare-associated pleural infection was consistently associated with poorer outcomes than community-acquired infection, with more comorbidities, multidrug-resistant organisms, and higher mortality [41].

Culture-negative infection, potentially reflecting prior antibiotics or limitations of conventional microbiological methods, did not consistently differ in outcome from culture-positive infection after adjustment for disease severity and patient characteristics [24,35].

Overall, bacteremia, healthcare-associated infection, and polymicrobial or antimicrobial-resistant infections showed the most consistent associations with poorer outcomes, whereas pleural fluid culture positivity alone was less consistently prognostic [18,24,27,30,34,35,41].

#### 3.3.6. Radiological and Procedural Predictors

Radiological and procedural characteristics were frequently evaluated as potential prognostic factors in adults with pleural infection. The most commonly investigated variables included pleural loculations, pleural thickening, extent of pleural involvement, timing of drainage or surgery, adequacy of pleural drainage, and surgical approach.

Pleural loculations were consistently associated with greater disease complexity and treatment failure; multiloculated collections more often required escalation, addi-tional pleural procedures, or surgery than free-flowing effusions [15,31]. Extensive pleural organization and radiological evidence of advanced disease were associated with prolonged hospitalization and poorer outcomes [19,42].

Delayed drainage or surgical referral was associated with more complications, longer hospitalization, and poorer outcomes, whereas earlier intervention, particularly timely surgery in selected patients, was associated with shorter hospital stay and better recovery [17,22,27,43].

Inadequate chest tube drainage was associated with prolonged hospitalization and greater need for additional drainage or surgery, and was identified in several co-horts as an independent predictor of treatment failure and persistent infection [18,31].

Among surgical patients, greater pleural thickening, advanced organization, de-layed referral, and elevated pleural LDH were associated with conversion from VATS to open thoracotomy or more complex surgery [19,43]. Comparisons of VATS and thoracotomy were mainly descriptive of treatment strategies rather than assessments of independent prognostic factors.

Overall, pleural loculations, delayed intervention, inadequate drainage, and advanced pleural organization were the radiological and procedural factors most consistently associated with treatment failure, prolonged hospitalization, and surgical intervention [15,17,18,19,22,27,31,42,43].

### 3.4. Meta-Analysis

Where at least three studies reported comparable effect estimates for the same prognostic factor and clinical outcome, quantitative synthesis was performed using random-effects meta-analysis to account for anticipated clinical and methodological heterogeneity. Pooled effect estimates were expressed as odds ratios (ORs), hazard ratios (HRs), risk ratios (RRs), or mean differences (MDs), as appropriate, together with their corresponding 95% confidence intervals (CIs). Statistical heterogeneity was assessed using Cochran’s Q test, the I^2^ statistic, and τ^2^. Prognostic factors for which quantitative synthesis was not feasible because of insufficient or heterogeneous data were summarized narratively.

#### 3.4.1. RAPID Score and Mortality

Five studies including 877 participants reported directly extractable mortality data at 3 months or 90 days according to RAPID risk category [8,22,27,32,35]. For the primary meta-analysis, patients in the high-risk category (RAPID score 5–7) were compared with those in the combined low- and intermediate-risk categories (RAPID score 0–4). Mortality occurred in 57 of 192 high-risk patients (29.7%) and in 44 of 685 low/intermediate-risk patients (6.4%).

**Figure 2:**
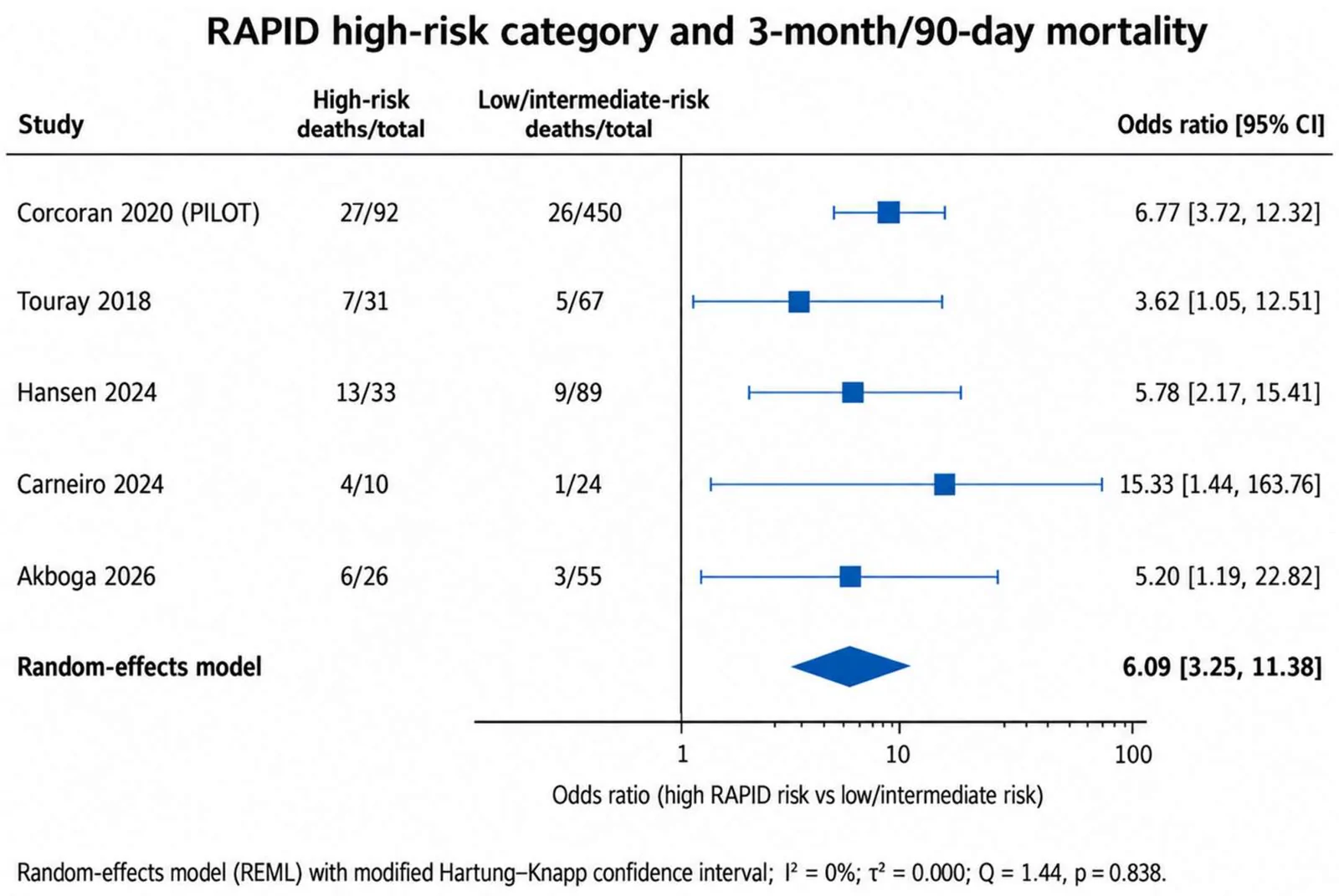
Association between high RAPID risk category and short-term mortality in adults with pleural infection. Corcoran 2020 (PILOT) [8]; Touray 2018 [36]; Hansen 2024 [27]; Carneiro 2024 [32]; Akboga 2026 [22].

Random-effects meta-analysis demonstrated that high RAPID risk was associated with significantly increased short-term mortality (pooled OR 6.09, 95% CI 3.25–11.38; *p* = 0.001). No significant between-study heterogeneity was observed (I^2^ = 0%; τ^2^ = 0.000; Q = 1.44, *p* = 0.838) (Figure 2).

Additional pairwise comparisons demonstrated a progressive increase in mortality across RAPID risk categories. Compared with the low-risk group, mortality was significantly higher in the intermediate-risk group (OR 3.23, 95% CI 1.07–9.77), the high-risk group compared with the intermediate-risk group (OR 4.02, 95% CI 2.04–7.91), and the high-risk group compared with the low-risk group (OR 11.62, 95% CI 3.73–36.18).

Detailed study-level data, including event counts, individual effect estimates, and supplementary analyses, are provided in Supplementary File S2, Table S3.

#### 3.4.2. Age and Short-Term Mortality

Three studies including 1239 participants reported adjusted odds ratios for age as a continuous predictor of short-term mortality and provided sufficiently comparable data for quantitative synthesis [18,23,35]. Chen et al. evaluated in-hospital mortality, Meyer et al. evaluated 90-day mortality, and Mwesige et al. evaluated in-hospital mortality. Effect estimates were standardized from odds ratios reported per 1-year increase in age to odds ratios per 10-year increase before pooling.

**Figure 3:**
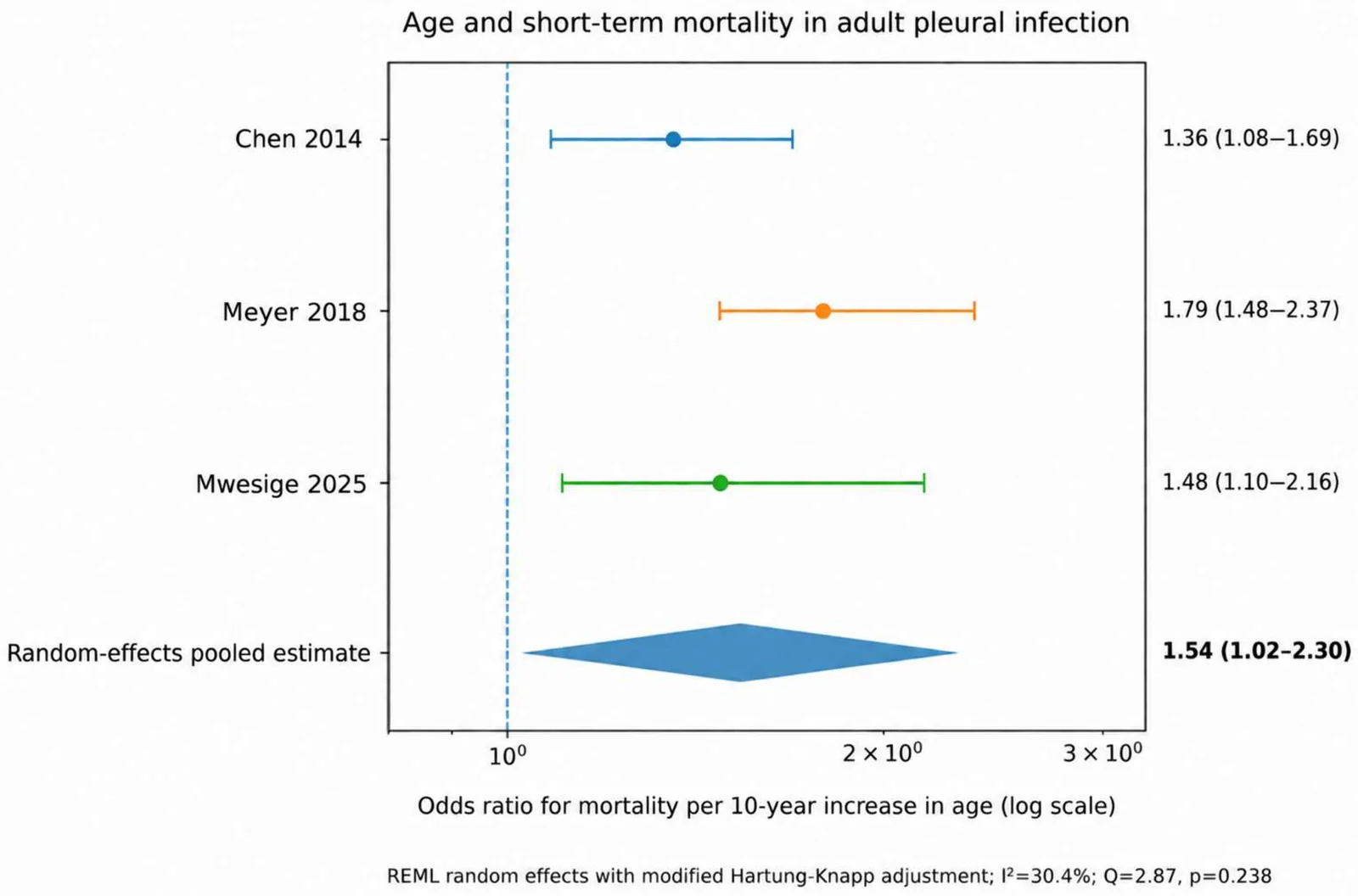
Association between age and short-term mortality in adults with pleural infection. Chen 2014 [18]; Meyer 2018 [35]; Mwesige 2025 [23].

Random-effects meta-analysis demonstrated that each 10-year increase in age was associated with significantly higher odds of short-term mortality (pooled OR 1.54, 95% CI 1.02–2.30; *p* = 0.045) (Figure 3). The estimated heterogeneity was I^2^ = 30.4% (τ^2^ = 0.009; Q = 2.87, *p* = 0.238); however, with only three contributing studies, Cochran’s Q has limited power, and these statistics do not exclude clinically important heterogeneity across the different mortality windows.

Studies reporting age using non-comparable categorical thresholds, unadjusted group summaries, or ambiguous effect estimates were not included in the quantitative synthesis. Detailed study-level estimates and data transformations are presented in Supplementary File S2, Table S4.

### 3.5. Sensitivity Analyses and Publication Bias

Sensitivity analyses were performed to evaluate the robustness of both quantitative syntheses.

**Figure 4:**
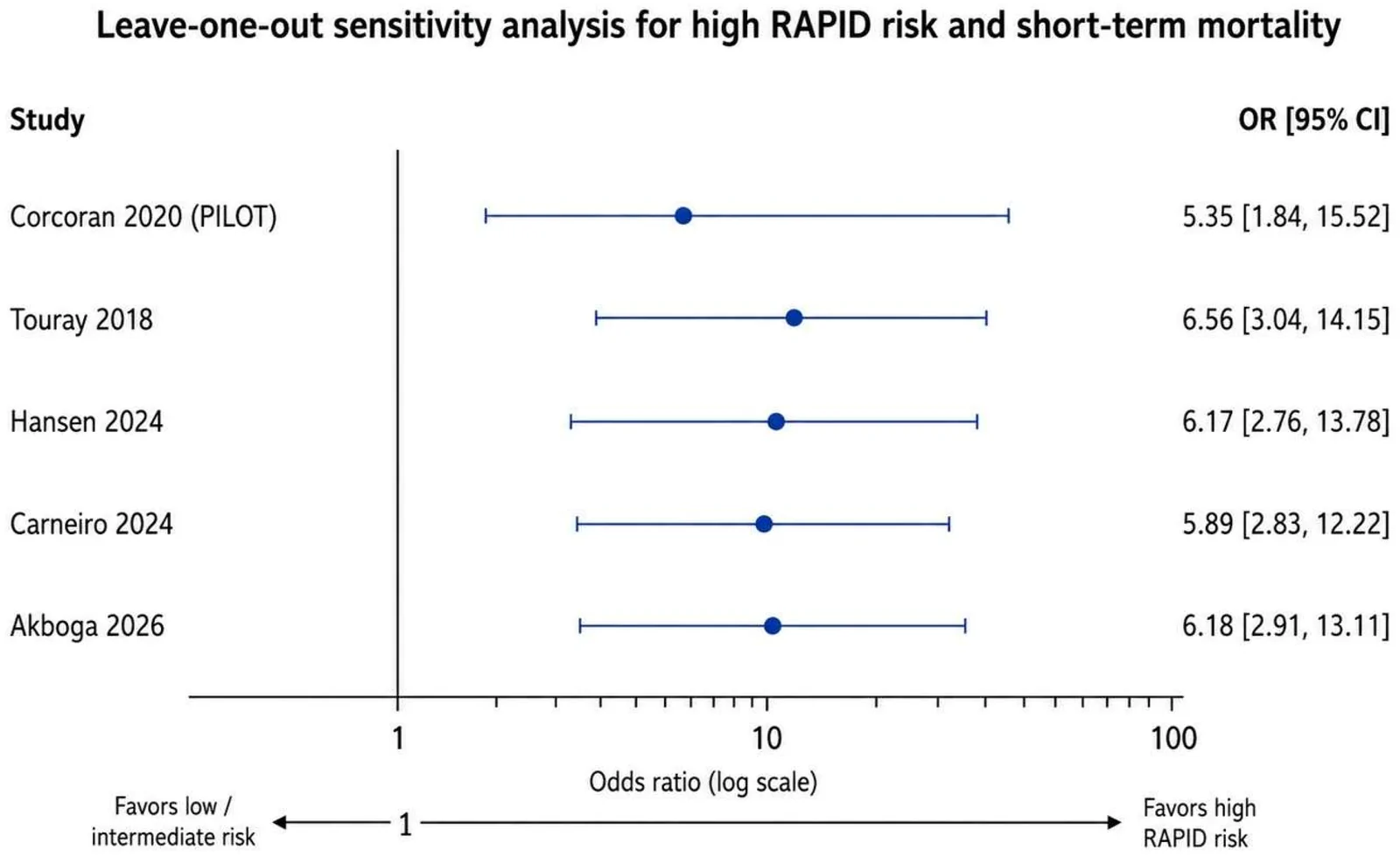
Leave-one-out sensitivity analysis of the association between high RAPID risk category and short-term mortality. Corcoran 2020 (PILOT) [8]; Touray 2018 [36]; Hansen 2024 [27]; Carneiro 2024 [32]; Akboga 2026 [22].

For the RAPID score meta-analysis, sequential leave-one-out analyses demonstrated that exclusion of individual studies did not materially alter the pooled effect estimate, with odds ratios ranging from 5.35 to 6.56, and all pooled estimates remaining statistically significant [8,22,27,32,36]. A fixed-effect model produced results comparable to those of the primary random-effects analysis (OR 6.09, 95% CI 3.91–9.47). Similarly, pooling risk ratios instead of odds ratios yielded a consistent association between high RAPID risk and short-term mortality (RR 4.52, 95% CI 2.71–7.55). Results of the leave-one-out analysis are presented in Figure 4.

For the age meta-analysis, a fixed-effect sensitivity analysis produced results comparable to those obtained with the primary random-effects model (OR per 10-year increase 1.53, 95% CI 1.33–1.78 versus OR 1.54, 95% CI 1.02–2.30), supporting the robustness of the pooled association [18,23,35].

Publication bias was not formally assessed because fewer than 10 studies were available for either quantitative synthesis, limiting the reliability of funnel plot interpretation and statistical tests for small-study effects. Similarly, subgroup analyses and meta-regression were not performed because of the limited number of studies included in each meta-analysis.

## 4. Discussion

This systematic review and meta-analysis synthesized evidence from 30 studies involving 8443 adults with complicated parapneumonic effusion, pleural empyema, or pleural infection. The principal quantitative finding was the strong association between the high-risk RAPID category and short-term mortality. Across five studies including 877 participants, high-risk patients had 4.52 times the risk of mortality at 3 months or 90 days compared with patients in the combined low- and intermediate-risk categories (pooled RR 4.52, 95% CI 2.71–7.55). The primary odds-ratio analysis yielded OR 6.09 (95% CI 3.25–11.38; I^2^ = 0%) [8,22,27,32,36]. Reporting both measures is important because mortality was not rare and the OR therefore overstates the association if interpreted as a risk ratio. This association remained robust across leave-one-out and fixed-effect analyses. Age was also associated with short-term mortality, but this estimate was less certain. Across only three studies including 1239 participants, each 10-year increase in age was associated with higher odds of mortality (pooled OR 1.54, 95% CI 1.02–2.30; I^2^ = 30.4%) [18,23,35] the studies combined in-hospital and 90-day mortality windows. Beyond these two quantitatively synthesized predictors, the available evidence suggests that comorbidities, laboratory biomarkers, pleural fluid characteristics, microbiological findings, and radiological and procedural factors may also contribute to prognostic assessment, although heterogeneity in predictor definitions, outcome measures, and reporting precluded additional meta-analyses.

The RAPID score was the most extensively investigated prognostic model and demonstrated the most consistent association with mortality. Its components—renal function, age, pleural fluid purulence, infection source, and serum albumin—are routinely available at presentation, enabling rapid risk stratification without specialized investigations. Our pooled estimate extends previous validation studies by quantifying the magnitude of mortality risk associated with the high-risk category. The observed association is consistent with earlier studies demonstrating a progressive increase in 3-month mortality across low-, intermediate-, and high-risk RAPID categories [8,37,73]. However, RAPID appears to be better established for predicting mortality than for identifying treatment failure or guiding therapeutic decision-making. Sedhai et al. reported limited discrimination of RAPID for treatment success following intrapleural enzyme therapy, whereas Simon et al. and Akboga et al. highlighted uncertainty regarding its ability to guide surgical selection [21,22,25]. Therefore, a high RAPID score should not be interpreted as an automatic indication for surgery or intrapleural therapy. Instead, it should prompt closer monitoring, early multidisciplinary assessment, and timely reassessment of treatment response. Recent evidence likewise recognizes RAPID as the most extensively validated prognostic model in pleural infection, while emphasizing the need for prospective impact studies to determine whether RAPID-guided management improves patient outcomes [73].

Crucially, RAPID is a prognostic score and has not been established as a predictor of benefit from a particular treatment. Several components, including age, renal dysfunction, and hypoalbuminemia, reflect baseline vulnerability that may drive mortality but may not be modifiable by more aggressive pleural intervention. Conversely, some patients at the highest predicted mortality risk may be frail or unsuitable for invasive treatment; therefore, a greater risk of death does not itself imply a greater absolute benefit from surgery or intrapleural therapy. The British Thoracic Society identified assessment of whether RAPID-directed care improves clinical outcomes as a research priority [3]. The RAPTOR-f randomized feasibility study is comparing usual care with RAPID-driven care and should clarify whether this strategy is acceptable and deliverable before a definitive outcome trial [74].

The pooled association between age and mortality was clinically plausible but should be interpreted cautiously. Only three studies contributed, the 95% confidence interval narrowly excluded the null, and the mortality windows differed between in-hospital and 90-day outcomes; with k = 3, heterogeneity statistics are also imprecise. Age is likely to reflect reduced physiological reserve, frailty, renal dysfunction, and cumulative comorbidity rather than acting as an isolated determinant of outcome. This interpretation is supported by the qualitative findings, in which chronic kidney disease, malignancy, immunosuppression, diabetes mellitus, and higher comorbidity scores were frequently associated with adverse outcomes [23,26,33,35,38,42]. Nevertheless, these associations were not uniform, and at least one surgical cohort found that advanced age alone was less informative after accounting for comorbidities and sepsis-related organ dysfunction [42]. A previous systematic review similarly reported a high burden of comorbidity, prolonged hospitalization, and substantial mortality among adults with pleural infection, while also highlighting that most available evidence originated from high-income countries [75]. These findings suggest that age should be interpreted in conjunction with comorbidity burden, organ dysfunction, and baseline functional status rather than as an isolated determinant of treatment intensity.

Routine laboratory markers provided additional prognostic information but could not generally be pooled. Hypoalbuminemia and elevated urea were repeatedly associated with poorer outcomes and are already incorporated into the RAPID score. Their independent prognostic effects were difficult to quantify because most studies reported them as components of multivariable prognostic models rather than as separate, consistently scaled predictors. The prognostic value of C-reactive protein (CRP) also depended on the method of assessment. Baseline CRP alone was not consistently associated with mortality, whereas serial increases appeared to provide greater prognostic information in the large database cohort reported by Shiroshita et al. [24]. This observation suggests that dynamic inflammatory changes during treatment may better reflect persistent infection and inadequate source control than a single baseline measurement. Hemoglobin, neutrophil fraction, leukocyte count, and other routine hematological indices were associated with mortality or prolonged hospitalization in selected cohorts, although the available evidence was too heterogeneous to support clinically applicable thresholds [18,34,43].

Pleural fluid, microbiological, and radiological variables appeared to capture complementary aspects of disease severity. Low pleural glucose, elevated pleural LDH, reduced pleural pH, pleural thickening, and pleural loculation were associated with recurrence, treatment failure, conversion to thoracotomy, or prolonged recovery in several studies [19,26,31,37]. However, the reported thresholds and outcome defini-tions varied considerably. Positive microbiological cultures and bacteremia were gen-erally associated with a more complicated clinical course, although their prognostic implications depended on the causative organism, acquisition setting, prior antimicro-bial exposure, and sampling method [20,28,29,35,40,41]. Wong et al. demonstrated that microbiologically positive pleural infection was independently associated with mor-tality and that selected pathogens were associated with particularly poor survival [28]. These findings are consistent with a previous systematic review reporting substantial geographic and setting-related variation in pleural microbiology, including differences between community- and hospital-acquired infections [76]. Accordingly, microbiolog-ical findings may contribute to prognostic assessment, but they should be interpreted in the context of local epidemiology and the limited sensitivity of conventional culture techniques.

Procedural and timing-related factors were also clinically relevant. Delayed pleu-ral drainage, delayed surgical referral, ineffective initial drainage, advanced pleural organization, and longer symptom duration were associated with treatment failure, conversion to open surgery, recurrence, or prolonged hospitalization [19,23,26,30,35,43]. Nevertheless, these variables are susceptible to confounding by in-dication, as patients with more advanced disease may both experience delayed clinical resolution and require more invasive treatment, making it difficult to distinguish an independent prognostic effect from underlying disease severity. A systematic review evaluating the timing of intervention in pleural infection reported a general trend to-ward shorter hospital stay, lower conversion rates, and fewer complications with ear-lier diagnosis and intervention, although no clear mortality benefit was demonstrated [77]. Similarly, a recent meta-analysis comparing surgery with intrapleural fibrinolysis reported higher treatment success and shorter hospital stay and chest-drain duration with surgery, without a significant difference in in-hospital mortality [78]. These find-ings provide important clinical context but should not be interpreted as evidence that any single prognostic factor identifies the optimal intervention for an individual pa-tient.

The clinical implications of the present findings are that prognostic assessment in pleural infection should be multidimensional. The RAPID score provides an immediate and standardized estimate of mortality risk, whereas age, comorbidity burden, serial inflammatory markers, microbiological findings, pleural fluid characteristics, imaging features, and early treatment response may further refine risk stratification. Patients at high risk may benefit from closer monitoring, early respiratory and thoracic surgical assessment, and prompt evaluation of source control. However, the current evidence does not establish that treatment escalation based solely on a high RAPID score or any individual biomarker improves survival. Clinical decision-making should continue to incorporate physiological reserve, frailty, bleeding risk, radiological stage, surgical suitability, patient preferences, and response to antibiotic therapy and pleural drainage [1,2,3,73]. This distinction is important because a prognostic factor may predict mortality without necessarily identifying patients who will benefit from a specific intervention.

The present review differs from previous evidence syntheses by integrating the broad spectrum of routinely available prognostic factors rather than addressing comorbidities, microbiology, treatment timing, prognostic scores, or treatment strategies in isolation. Previous systematic reviews have separately characterized the burden of comorbid disease [75], global microbiological patterns [76], the influence of treatment timing [77], and comparative outcomes of surgery and intrapleural fibrinolysis [78]. By integrating these domains within a single prognostic framework, the present review provides quantitative estimates for two clinically accessible predictors—the RAPID risk category and age. At the same time, the limited feasibility of additional meta-analyses highlights an important limitation of the current literature: many potentially relevant prognostic factors are reported using heterogeneous definitions, thresholds, follow-up periods, and effect measures, limiting quantitative synthesis and direct comparison across studies.

This review has several strengths. It was conducted using a predefined PECO framework, a comprehensive search of four major databases, and evidence from geographically diverse populations and varied clinical settings. Prognostic factors were organized into clinically meaningful domains, and quantitative synthesis was restricted to predictors with sufficiently comparable definitions and effect estimates. Random-effects meta-analysis, modified Hartung–Knapp confidence intervals, alternative effect measures, and leave-one-out analyses were used to evaluate the robustness of the pooled findings. The methodological quality of the included studies was assessed using the Newcastle–Ottawa Scale, allowing the findings to be interpreted in the context of the strengths and limitations of the available evidence.

Several limitations should nevertheless be acknowledged. Most included studies were retrospective and single-center, introducing risks of selection bias, residual confounding, incomplete data, and limited generalizability. Definitions of treatment failure varied substantially and included drainage failure, the need for intrapleural therapy, surgical escalation, persistent sepsis, recurrence, prolonged hospitalization, or mortality. The included studies spanned more than two decades, during which diagnostic imaging, antimicrobial therapy, drainage techniques, intrapleural enzyme therapy, and surgical practice evolved considerably. The RAPID meta-analysis pooled unadjusted event data and combined the low- and intermediate-risk categories into a single comparator group, improving statistical feasibility at the expense of clinical granularity. The age meta-analysis included only three studies and combined in-hospital with 90-day mortality, contributing to clinical heterogeneity and imprecision. Publication bias could not be formally assessed because fewer than 10 studies were available for either quantitative synthesis. Restriction to English-language full-text publications may have introduced language bias, and the possibility of unrecognized overlap between cohorts from the same institutions cannot be completely excluded. Finally, although the Newcastle–Ottawa Scale provides a standardized assessment of methodological quality in observational studies, it does not evaluate all aspects of prognostic model development, validation, calibration, or clinical utility.

Future research should prioritize prospective, multicenter cohorts with standardized measurement of prognostic factors at baseline and a consensus definition of treatment failure. Studies should report exact event counts, adjusted effect estimates with confidence intervals, predefined follow-up time points—particularly 30- and 90-day outcomes—and complete information on missing data. In addition to its discriminatory performance, the RAPID score should be evaluated for calibration, clinical utility, and incremental prognostic value beyond routine clinical assessment. Most importantly, prospective impact studies are needed to determine whether management strategies guided by RAPID or combined prognostic models improve patient-important outcomes [73]. Prognostic models integrating RAPID with serial inflammatory markers, microbiological findings, pleural fluid biochemistry, and imaging features may ultimately provide greater predictive accuracy but will require external validation and evaluation of their clinical feasibility before routine implementation. Overall, the available evidence supports the RAPID score and age as useful predictors of mortality risk, while emphasizing that prognostic assessment should remain multidimensional, dynamic, and complementary to individualized clinical judgment.

## 5. Conclusions

This systematic review and meta-analysis demonstrates that routinely available clinical information can provide meaningful prognostic stratification in adults with complicated parapneumonic effusion, pleural empyema, or pleural infection. Among the evaluated prognostic factors, the RAPID score showed the most consistent association with short-term mortality. Patients in the high-risk RAPID category had more than four times the risk of death at 3 months or 90 days compared with those in the combined low- and intermediate-risk categories (RR 4.52, 95% CI 2.71–7.55; corresponding OR 6.09, 95% CI 3.25–11.38). Increasing age may also be prognostic, but the pooled estimate was derived from only three studies, combined different mortality windows, and had a confidence interval that only narrowly excluded the null; it should therefore be interpreted as less certain than the RAPID finding.

Several additional routinely available factors—including comorbidity burden, renal dysfunction, hypoalbuminemia, serial inflammatory marker trajectories, bacteremia, microbiological findings, pleural fluid glucose and LDH, pleural thickening, pleural loculation, and delayed or ineffective pleural drainage—may further refine prognostic assessment. However, heterogeneity in predictor definitions, outcome measures, follow-up intervals, and statistical reporting limited quantitative synthesis for most of these variables.

The available evidence supports the use of the RAPID score as an adjunct to, rather than a replacement for, comprehensive clinical assessment. Prognostic information should be interpreted alongside comorbidity burden, physiological reserve, microbiological and radiological findings, and early response to treatment. Future prospective multicenter studies using standardized definitions of treatment failure, uniform reporting of adjusted effect estimates, and predefined follow-up intervals are needed to validate integrated prognostic models and determine whether risk-guided management improves patient-important outcomes.

## Figures and Tables

**Table 1:** Summary of Included Studies.

Study ID	Country	Population	Sample Size	Study Design	Routinely Available Predictors	Comparator/Groups	Primary and Secondary Outcomes	Main Prognostic Findings	Follow-up/Synthesis
**Kim 2024 [20]**	South Korea	Adults with complicated pleural infection requiring pleural drainage.	341	Retrospective cohort	Bacteremia, RAPID score, cavitary lesion, pleural fluid culture positivity.	Bacteremia vs. no bacteremia; RAPID score levels.	In-hospital mortality and overall mortality.	Bacteremia was independently associated with higher mortality; increasing RAPID score was strongly associated with overall mortality.	In-hospital and longitudinal mortality; meta-analysis candidate for RAPID/mortality.
**Misthos 2005 [16]**	Greece	Adults with postpneumonic empyema/complicated parapneumonic effusion.	127	Prospective randomized study with prognostic analysis	Loculations, drainage completeness, pleural fluid and baseline clinical features.	Chest tube drainage alone vs. early intrapleural fibrinolysis.	Treatment success/failure, need for surgery, hospital stay, mortality.	Early fibrinolysis improved treatment success; loculations, drainage completeness, and baseline features predicted outcome.	In-hospital outcomes; narrative/treatment-failure synthesis.
**Simon 2023 [21]**	France	Patients managed for pleural infection in a thoracic surgery department.	74	Single-center retrospective cohort	RAPID score; surgery status.	Low-, medium-, and high-risk RAPID groups; medical vs. surgical management.	Survival at 6 and 12 months; length of stay; surgical intervention.	High-risk RAPID classification was associated with significantly lower 6- and 12-month survival.	6 and 12 months; meta-analysis candidate for RAPID/mortality.
**Kim 2014 [17]**	South Korea	Adults admitted with pleural infection treated initially with medical management.	127	Retrospective cohort	Age, symptom duration, smoking, serum albumin, pleural glucose, pleural LDH.	Medical treatment success vs. medical treatment failure.	Failure of medical treatment requiring additional surgical treatment.	Younger age and shorter symptom duration independently predicted failure of initial medical management requiring surgery.	In-hospital treatment outcome; meta-analysis candidate for treatment failure.
**Davies 1999 [15]**	United Kingdom	Consecutive patients with pleural infection receiving chest tube drainage and antibiotics.	85	Prospective cohort	Clinical, radiological, microbiological and pleural fluid predictors, including purulence.	Medical treatment success vs. failure.	Medical treatment failure requiring surgery and long-term survival.	Absence of frank purulence predicted medical-treatment success; baseline clinical, radiological, and pleural-fluid variables were associated with treatment failure and long-term survival.	Long-term survival; meta-analysis/narrative synthesis.
**Akboga 2026 [22]**	Turkey	Adults diagnosed with pleural empyema in a thoracic surgery clinic.	81	Retrospective single-center cohort	RAPID score and components; surgical timing/eligibility variables.	Surgical decortication vs. tube thoracostomy; RAPID risk groups.	Survival, surgical treatment, complications, length of stay.	Higher RAPID scores were associated with poorer short-term survival, although treatment-selection bias may have influenced the association.	In-hospital/short-term outcomes; narrative/RAPID synthesis.
**Chen 2014 [18]**	Taiwan	Patients with acute thoracic empyema undergoing outcome analysis of VATS.	602	Retrospective cohort	Age, comorbidities, microbiology, culture positivity/polymicrobial infection, treatment modality.	VATS vs. non-operative drainage/management; survivor vs. non-survivor outcomes.	In-hospital mortality and treatment outcomes.	Older age, multimorbidity, polymicrobial infection, and microbiological features were associated with poorer in-hospital outcomes.	In-hospital outcomes; narrative/possible mortality synthesis.
**Mwesige 2025 [23]**	Uganda	Adults with empyema thoracis managed at Mulago National Referral Hospital.	200	Retrospective cohort	Age, etiology, delay to drainage, comorbidities, clinical parameters.	Parapneumonic vs. non-parapneumonic etiology; delayed vs. early drainage.	In-hospital mortality and need for re-intervention.	Older age, non-parapneumonic etiology, and delayed drainage independently predicted mortality and re-intervention.	In-hospital outcomes; narrative/mortality and re-intervention synthesis.
**Shiroshita 2025 [24]**	Japan	Adults aged ≥ 40 years with pleural empyema undergoing thoracostomy in a real-world database.	823	Database cohort/longitudinal survival analysis	Baseline and serial serum CRP levels.	Baseline CRP and serial CRP trajectory groups.	Death within 90 days; thoracic surgery as competing risk.	Baseline CRP was not prognostic, whereas persistently rising serial CRP independently predicted higher 90-day mortality.	90 days; meta-analysis candidate for serial CRP if comparable.
**Sedhai 2025 [25]**	United States	Adults with complex pleural effusion/empyema treated with intrapleural tPA/DNase.	55	Single-center retrospective observational study	RAPID score, comorbidities, clinical variables.	Treatment success vs. failure; survivors vs. non-survivors.	Treatment success without surgery, bleeding, ICU escalation, mortality at 30 days and 6 months.	RAPID showed limited ability to predict treatment success or 6-month mortality after intrapleural tPA/DNase.	30 days and 6 months; narrative/IPFT failure synthesis.
**Liu 2025 [26]**	Taiwan	Adults with bacterial empyema after surgical treatment.	1000	Retrospective cohort	Diabetes, pleural glucose, Streptococcus infection, delay to surgery and other clinical factors.	Recurrent vs. non-recurrent empyema.	Empyema recurrence and associated adverse outcomes.	Diabetes, low pleural glucose, Streptococcus infection, and delayed surgery independently predicted recurrent empyema.	Recurrence outcome; narrative synthesis.
**Hansen 2024 [27]**	Denmark	Patients admitted with pleural infection at a regional university hospital.	122	Retrospective observational cohort	RAPID score and components.	Low-, medium-, and high-risk RAPID groups.	3-month mortality, 12-month mortality, length of stay, initial treatment failure, surgical intervention.	Higher RAPID category was associated with greater mortality, longer hospitalization, and treatment failure.	3 and 12 months; meta-analysis candidate for RAPID/mortality.
**Wong 2025 [28]**	Hong Kong	Adults hospitalized for pleural infection in two acute-care hospitals.	381	Bicentric retrospective observational cohort	Pleural fluid microbiological positivity, specific organisms, RAPID score.	Microbiological-positive vs. microbiological-negative pleural infection.	Mortality at 3 months and long-term time points; surgery, ICU, length of stay.	Pleural-fluid microbiological positivity independently predicted mortality; S. aureus and non-Enterobacteriaceae Gram-negative organisms predicted poorer survival.	3 months to long-term follow-up; meta-analysis candidate for microbiology/mortality.
**Liu 2025 [29]**	Taiwan	Adults with stage II/III bacterial empyema treated surgically.	225	Retrospective cohort	Respiratory secretion culture, pleural fluid/tissue cultures, microbiology.	Culture-positive vs. culture-negative/respiratory secretion culture groups.	Surgical outcome, ICU stay, hospital stay, mortality.	Positive respiratory-secretion cultures were associated with poorer surgical outcomes and prolonged recovery.	Postoperative outcomes; narrative/microbiology synthesis.
**Yong 2024 [30]**	Singapore	Patients with pleural infection treated with intrapleural fibrinolytic therapy.	131	Retrospective cohort	Age, RAPID score, radiological features, timing and dose of IPFT.	Treatment success vs. failure; different alteplase dosing/timing groups.	Treatment success, defined as survival without surgery at 3 months.	Earlier fibrinolytic therapy and younger age independently predicted treatment success without surgery.	3 months; narrative/IPFT treatment-failure synthesis.
**Khemasuwan 2025 [31]**	United States	Patients receiving intrapleural enzyme therapy for CPPE/empyema across five institutions.	466	Multicenter retrospective cohort with machine-learning prediction models	Pleural fluid LDH, pleural thickening, abscess/necrotizing pneumonia, clinical/radiological variables.	IET success vs. IET failure.	Failure of intrapleural tPA/DNase requiring escalation/surgery.	Machine-learning models identified pleural LDH, pleural thickening, lung abscess/necrotizing pneumonia, and radiological features as predictors of enzyme-therapy failure.	Treatment failure; narrative/possible meta-analysis if extractable.
**Carneiro 2024 [32]**	Brazil	Patients with stage III pleural empyema undergoing pulmonary decortication.	34	Retrospective cohort	RAPID score.	Low-, medium-, and high-risk RAPID groups.	3-month mortality, length of stay, readmission, pleural re-intervention.	High-risk RAPID classification was associated with increased 3-month mortality after decortication.	3 months; RAPID/mortality synthesis.
**Stüben 2023 [33]**	Germany	Patients surgically treated for pleural empyema at a university medical center.	225	Retrospective cohort	RAPID score, diabetes, renal replacement therapy, age, urea, albumin, blood cultures.	Original RAPID score vs. adjusted RAPID score with additional comorbidities.	90-day mortality and postoperative outcomes.	RAPID independently predicted 90-day mortality; adding diabetes and renal replacement therapy improved prognostic performance.	90 days; RAPID/mortality synthesis.
**Corcoran 2020 [8]**	International multicenter	Adults with pleural infection recruited prospectively in the PILOT study.	546	Prospective observational validation cohort	RAPID score and components.	Low-, medium-, and high-risk RAPID groups.	3-month mortality; 12-month mortality; length of stay; surgery; medical treatment failure; lung function.	RAPID stratified 3- and 12-month mortality risk and remained the best-validated prognostic model.	3 and 12 months; main meta-analysis candidate for RAPID.
**Tsai 2019 [34]**	Taiwan	Adult emergency department patients with thoracic empyema after VATS.	380	Retrospective cohort	Clinical, laboratory and thoracentesis-related factors.	Adverse outcome vs. non-adverse outcome groups.	In-hospital mortality and reintervention/adverse postoperative outcomes.	Routine clinical, laboratory, and thoracentesis-related variables independently predicted adverse postoperative outcomes after VATS.	Postoperative/in-hospital outcomes; narrative/mortality synthesis.
**Meyer 2018 [35]**	Denmark	Consecutive patients with pleural infection in nine hospitals.	437	Retrospective multicenter cohort	Etiology, comorbidity, drainage delay, surgical referral, intrapleural fibrinolysis, microbiology.	Outcome groups by mortality/ICU/surgical referral.	30- and 90-day mortality, ICU admission, surgical referral and treatment outcomes.	Etiology, comorbidity burden, delayed drainage, and treatment-related factors were associated with mortality and adverse outcomes.	30 and 90 days; narrative/mortality synthesis.
**Touray 2018 [36]**	United States	Patients with complicated parapneumonic effusions and empyema.	98	Retrospective cohort	RAPID score.	Low-, medium-, and high-risk RAPID groups.	90-day mortality, length of stay and healthcare cost.	Higher RAPID categories were associated with greater 90-day mortality, longer hospitalization, and higher costs.	90 days; RAPID/mortality synthesis.
**Wu 2018 [38]**	Taiwan	Patients with thoracic empyema.	165	Retrospective cohort	Charlson Comorbidity Index, CHADS2, CHA2DS2-VASc scores.	Risk-score strata.	Mortality in thoracic empyema.	Charlson Comorbidity Index, CHADS2, and CHA2DS2-VASc scores showed prognostic value for mortality.	Mortality follow-up; narrative/comorbidity synthesis.
**Park 2016 [40]**	South Korea	Patients with pleural infection/parapneumonic effusion in a single-center cohort.	421	Retrospective cohort	Microbiological characteristics, pathogens and clinical variables.	Survivors vs. non-survivors; microbiological groups.	Mortality in pleural infection.	Microbiological characteristics and routine clinical variables independently predicted mortality.	Mortality outcome; microbiology/mortality synthesis.
**Koma 2017 [41]**	Japan	Patients with community-acquired, health-care-associated or hospital-acquired empyema.	81	Retrospective cohort	Acquisition category, comorbidities, cancer, CNS disease, microbiology.	CAE vs. HCAE vs. HAE.	Mortality and treatment outcomes.	Healthcare-associated and hospital-acquired empyema were associated with higher mortality than community-acquired disease.	150-day survival curves; narrative/mortality synthesis.
**Kim 2016 [43]**	South Korea	Patients with community-acquired pneumonia and complicated parapneumonic effusion requiring chest tube drainage.	158	Retrospective cohort	Fever, PaO2, hemoglobin, blood neutrophil fraction, microbiological identification, ineffective drainage.	Shorter vs. prolonged hospitalization.	Prolonged hospital stay; drainage duration; surgery/decortication; mortality.	Fever, hypoxemia, anemia, elevated neutrophil fraction, microbiological identification, and ineffective drainage independently predicted prolonged hospitalization.	Hospital stay; meta-analysis candidate for LOS if comparable.
**Schweigert 2016 [42]**	Germany	Patients undergoing surgery for parapneumonic pleural empyema.	335	Retrospective surgical cohort	Charlson comorbidity score, ASA grade, pulmonary sepsis, respiratory failure, acute renal failure, age.	Mortality vs. survival; comorbidity and age strata.	Postoperative mortality, ICU care and postoperative morbidity.	Charlson Comorbidity Index ≥ 3, higher ASA grade, pulmonary sepsis, respiratory failure, and acute renal failure independently predicted postoperative mortality.	Postoperative outcomes; narrative/mortality synthesis.
**Tantraworasin 2018 [39]**	Thailand	Patients with stage II/III bacterial empyema thoracis.	382	Retrospective cohort	Sepsis status, decortication, persistent pleural space and clinical factors.	Recurrent vs. non-recurrent empyema.	Recurrent empyema thoracis.	Absence of preoperative sepsis, no decortication, and persistent pleural space independently predicted recurrence.	Long-term recurrence; narrative synthesis.
**Ahn 2018 [37]**	South Korea	Patients with pyogenic empyema undergoing VATS decortication.	221	Retrospective cohort	Diabetes, bacteremia, visceral pleural thickness, age, COPD, steroid use.	Groups by postoperative lung expansion/drainage duration.	Postoperative lung expansion, chest tube drainage duration and postoperative hospital stay.	Diabetes, bacteremia, and greater visceral pleural thickness independently predicted delayed lung expansion and prolonged recovery.	Postoperative outcomes; narrative synthesis.
**Salim 2016 [19]**	Egypt	Adults with assumed stage II postpneumonic empyema undergoing VATS debridement at a cardiothoracic surgery department.	120	Prospective study with prognostic analysis	Duration of symptoms before surgery, pleural fluid LDH, pleural thickness on CT, Gram-negative pleural infection, age, sex, diabetes, hypertension, liver disease, kidney disease, pleural pH, pleural glucose, pleural protein, leukocytosis.	Successful VATS management vs. conversion to open thoracotomy.	Conversion from VATS to thoracotomy; postoperative complications; 30-day mortality.	Longer symptom duration, Gram-negative infection, higher pleural LDH, and greater pleural thickness independently predicted conversion to thoracotomy.	Perioperative outcomes; narrative surgical treatment-failure synthesis.

Abbreviations: CAE, community-acquired empyema; CNS, central nervous system; COPD, chronic obstructive pulmonary disease; CPPE, complicated parapneumonic pleural effusion; HAE, hospital-acquired empyema; HCAE, health-care-associated empyema; ICU, intensive care unit; IET, intrapleural enzyme therapy; IPFT, intrapleural fibrinolytic therapy; LDH, lactate dehydrogenase; LOS, length of stay; RAPID, Renal, Age, Purulence, Infection source, Dietary factors; tPA/DNase, tissue plasminogen activator/deoxyribonuclease; VATS, video-assisted thoracoscopic surgery.

## Data Availability

The original contributions presented in this study are included in the article and the Supplementary Files. Further inquiries can be directed to the corresponding author.

## Supplementary Materials

The following supporting information can be downloaded at: https://www.mdpi.com/article/10.3390/jcm15207778/s1, File S1: Full Search Strategies; File S2: Table S1. Excluded Studies and Reasons for Exclusion; Table S2. Quality Assessment of Included Articles using the Newcastle-Ottawa Scale; Table S3. Study-Level Data Used in the Meta-Analysis of RAPID Score and Short-Term Mortality; Table S4. Study-Level Data Used in the Meta-Analysis of Age and Short-Term Mortality; File S3: PRISMA 2020 for Abstract Checklist.

## Abbreviations

The following abbreviations are used in this manuscript:

ASA	American Society of Anesthesiologists
CAE	Community-acquired empyema
CCI	Charlson Comorbidity Index
CI	Confidence interval
CNS	Central nervous system
COPD	Chronic obstructive pulmonary disease
CPE	Complicated parapneumonic effusion
CPPE	Complicated parapneumonic pleural effusion
CRP	C-reactive protein
CT	Computed tomography
CRediT	Contributor Roles Taxonomy
DNase	Deoxyribonuclease
HAE	Hospital-acquired empyema
HCAE	Healthcare-associated empyema
HR	Hazard ratio
ICU	Intensive care unit
IET	Intrapleural enzyme therapy
IPFT	Intrapleural fibrinolytic therapy
LDH	Lactate dehydrogenase
LOS	Length of hospital stay
MD	Mean difference
MeSH	Medical Subject Headings
MNPI	Microbiological-negative pleural infection
MPPI	Microbiological-positive pleural infection
NLR	Neutrophil-to-lymphocyte ratio
NOS	Newcastle–Ottawa Scale
OR	Odds ratio
PECO	Population, Exposure, Comparator, Outcome
PRISMA	Preferred Reporting Items for Systematic Reviews and Meta-Analyses
PROSPERO	International Prospective Register of Systematic Reviews
Q	Cochran’s Q statistic
RAPID	Renal function, Age, Purulence, Infection source, and Dietary factors
REML	Restricted maximum likelihood
RR	Risk ratio
SD	Standard deviation
tPA	Tissue plasminogen activator
VATS	Video-assisted thoracoscopic surgery
I^2^	Higgins’ inconsistency statistic
τ^2^	Between-study variance
