# Supplementary material for: Routinely Available Prognostic Factors for Adverse Outcomes in Adults with Complicated Parapneumonic Effusion and Empyema: A Systematic Review and Meta-Analysis
Source: J Clin Med. 2026 Oct 9;15(20):7778. doi: 10.3390/jcm15207778 (PMC13705253; doi:10.3390/jcm15207778)
Supplement: Supplementary file 1 [file jcm-15-07778-s001.zip › Supplementary File S1 Search Strategy.pdf]

Routinely Available Prognostic Factors for Treatment Failure in Adults with Complicated Parapneumonic Effusion and Em-pyema: A Systematic Review and Meta-analysis

Supplementary File S1. Full Search Strategies.

The literature search was conducted in four electronic databases: PubMed, Scopus, Web of Science, and the Cochrane Library. The search strategy was designed to identify original human clinical studies evaluating routinely available prognostic factors associated with treatment failure, mortality, and other adverse clinical outcomes in adults with complicated parapneumonic effusion, pleural empyema, or pleural infection.
The search combined controlled vocabulary, where available, with free-text terms related to pleural empyema, pleural infection, complicated parapneumonic effusion, prognosis, risk factors, predictive factors, RAPID score, treatment failure, drainage failure, surgical referral, video-assisted thoracoscopic surgery, decortication, and mortality. Full reproducible search strategies for each database are provided below. The last search was performed on 06 July 2026.

1. Search Strategy Development

The search strategy was developed according to the PECO (Population, Exposure, Comparator, Outcome) framework adopted for this systematic review and meta-analysis. The population component included adults with complicated parapneumonic effusion, pleural empyema, or pleural infection. The exposure component included routinely available clinical, biochemical, microbiological, and pleural fluid prognostic factors, including but not limited to age, comorbidities, RAPID score, C-reactive protein, albumin, urea, neutrophil-to-lymphocyte ratio, pleural fluid pH, pleural fluid glucose, pleural fluid lactate dehydrogenase, pleural purulence, loculations, bacteremia, and microbiological findings. The outcome component included treatment failure and related adverse clinical outcomes, such as failure of chest tube drainage, need for treatment escalation, need for surgery, video-assisted thoracoscopic surgery, decortication, mortality, intensive care unit admission, readmission, recurrence, and prolonged length of hospital stay.
In PubMed, Medical Subject Headings were combined with free-text terms. In Scopus, the search was conducted in title, abstract, and keyword fields using the TITLE-ABS-KEY function. In Web of Science, topic-based free-text terms were combined using Boolean operators. In the Cochrane Library, equivalent keyword combinations were used, including proximity operators where applicable.
The search was limited to studies involving adult human populations. Case reports, pediatric studies, tuberculous pleural disease, malignant pleural effusion, and non-target populations were excluded using database filters and exclusion terms where possible. In addition to database searching, the reference lists of included studies and relevant reviews were manually screened to identify additional eligible studies.

2. Full Database Search Strategies

Table S1 presents the number of records retrieved from each database and the exact search queries used for the systematic review.

Search Engine	Number of Records Found	Exact Search Query
**Pubmed**	48	(("Diabetes Mellitus, Type 2"[MeSH] OR "type 2 diabetes" OR "type 2 diabetes mellitus" OR T2DM OR T2D)

		AND<br>("Sodium-Glucose Transporter 2 Inhibitors"[MeSH] OR "SGLT2 inhibitor*" OR "SGLT-2 inhibitor*" OR SGLT2i OR SGLT2 OR empagliflozin OR dapagliflozin OR canagliflozin OR ertugliflozin)<br>AND<br>("left ventricular global longitudinal strain" OR "global longitudinal strain" OR "LV GLS" OR LVGLS OR GLS OR "left ventricular strain" OR "myocardial strain" OR "speckle-tracking echocardiography" OR "speckle tracking echocardiography" OR "strain echocardiography"))
**Scopus**	54	TITLE-ABS-KEY (<br>("type 2 diabetes" OR "type 2 diabetes mellitus" OR T2DM OR T2D)<br>AND<br>("SGLT2 inhibitor*" OR "SGLT-2 inhibitor*" OR SGLT2i OR SGLT2 OR empagliflozin OR dapagliflozin OR canagliflozin OR ertugliflozin)<br>AND<br>("left ventricular global longitudinal strain" OR "global longitudinal strain" OR "LV GLS" OR LVGLS OR GLS OR "left ventricular strain" OR "myocardial strain" OR "speckle-tracking echocardiography" OR "speckle tracking echocardiography" OR "strain echocardiography")<br>)
**Web of Science**	129	("type 2 diabetes" OR "type 2 diabetes mellitus" OR T2DM OR T2D) AND ("SGLT2 inhibitor*" OR "SGLT-2 inhibitor*" OR SGLT2i OR SGLT2 OR empagliflozin OR dapagliflozin OR canagliflozin OR ertugliflozin) AND ("left ventricular global longitudinal strain" OR "global longitudinal strain" OR "LV GLS" OR LVGLS OR GLS OR "left ventricular strain" OR "myocardial strain" OR "speckle-tracking echocardiography" OR "speckle tracking echocardiography" OR "strain echocardiography")
**Cochrane library**	69	("type 2 diabetes" OR "type 2 diabetes mellitus" OR T2DM OR T2D) AND ((SGLT2 NEXT inhibitor*) OR (SGLT-2 NEXT inhibitor*) OR SGLT2i OR SGLT2 OR empagliflozin OR dapagliflozin OR canagliflozin OR ertugliflozin) AND ("left ventricular global longitudinal strain" OR "global longitudinal strain" OR "LV GLS" OR LVGLS OR GLS OR "left ventricular strain" OR "myocardial strain" OR "speckle tracking echocardiography" OR "strain echocardiography")
