# Supplementary material for: Routinely Available Prognostic Factors for Adverse Outcomes in Adults with Complicated Parapneumonic Effusion and Empyema: A Systematic Review and Meta-Analysis
Source: J Clin Med. 2026 Oct 9;15(20):7778. doi: 10.3390/jcm15207778 (PMC13705253; doi:10.3390/jcm15207778)
Supplement: Supplementary file 1 [file jcm-15-07778-s001.zip › Supplementary File S2.pdf]

Routinely Available Prognostic Factors for Treatment Failure in Adults with Complicated Parapneumonic Effusion and Empyema: A Systematic Review and Meta-analysis
Supplementary File S2

Table S1. Excluded Studies and Reasons for Exclusion.

**Grendelmeier 2019 [DOI: 10.1055/s-0039-1695058]**	The study was excluded because it is a narrative review/management article on pleural effusion clinical scoring systems. Although it discusses RAPID and other pleural disease scores, it does not include an original cohort or provide extractable predictor-outcome data for adults with complicated parapneumonic effusion or empyema.
**Höfken 2018 [DOI: 10.1055/a-0648-0145]**	The study was excluded because it appears to focus on standardized surgical/VATS management of parapneumonic empyema and does not clearly report an eligible association between a routinely available prognostic factor and treatment failure, mortality, or another relevant adverse outcome.
**Park 2025 [DOI: 10.1016/j.jiac.2025.102722]**	The study was excluded because it compares community-acquired primary pleural infection with postpneumonic pleural infection. Although it reports clinical, microbiological, radiological, and outcome data, the main analysis identifies factors associated with infection subtype rather than prognostic factors associated with treatment failure or adverse outcomes.
**Sziklavari 2025 [PMID: 39178929]**	The study was excluded because it evaluates outcomes of vacuum-assisted closure in patients with empyema thoracis. The focus is a therapeutic/procedural approach, without a clear analysis of routinely available prognostic factors associated with treatment failure, mortality, or other eligible adverse outcomes.
**Gonnelli 2025 [DOI: 10.1186/s12931-025-03391-7]**	The study was excluded because it primarily evaluates management strategy or procedural referral patterns in pleural infection. It does not clearly report an eligible routinely available predictor-outcome association for treatment failure or mortality.
**Yiu 2025 [DOI: 10.1111/imj.70028]**	The study was excluded because it evaluates the impact of a specialised pleural medicine unit on the management of pleural infection. The intervention is organisational/management-based and does not focus on routinely available prognostic factors associated with treatment failure or adverse outcomes.
**Shen 2014 [DOI: 10.1620/tjem.232.285]**	The study was excluded because the main exposure is institutional caseload or hospital volume rather than a routinely available patient-level clinical, biochemical, microbiological, or pleural fluid prognostic factor.
**Schweigert 2012 [DOI: 10.1308/003588412X13171221592212]**	The study was excluded because it focuses on surgical management of empyema in very elderly patients and does not clearly provide an eligible analysis of routinely available prognostic factors associated with treatment failure or mortality.
**Klopp 2008 [DOI: 10.1007/s00104-007-1429-y]**	The study was excluded because it is a management/overview article on pleural empyema treatment rather than an original prognostic study with extractable predictor-outcome data.
**Yang 2017 [DOI: 10.21037/jtd.2017.10.96]**	The study was excluded because it is a review/management article on infectious pleural effusion and does not provide original cohort data or extractable prognostic factor-outcome associations.

**Falguera 2011 [DOI: 10.1183/09031936.00000211]**	The study was excluded because it evaluates predictors for the development of complicated parapneumonic effusion or empyema among patients with pneumonia. The population is not restricted to adults already diagnosed with complicated parapneumonic effusion or empyema, and the outcome is disease occurrence rather than treatment failure.
**Kolditz 2008 [DOI: 10.1007/s10405-008-0248-2]**	The study was excluded because it is an overview/management article on parapneumonic effusion and empyema, without original prognostic factor analysis or extractable predictor-outcome data.
**Dean 2016 [DOI: 10.1016/j.chest.2015.12.027]**	The study was excluded because it evaluates pleural effusion as a predictor of poor outcomes in patients presenting with pneumonia. It does not specifically study adults with established complicated parapneumonic effusion or empyema as the target population.
**Nwagboso 2022 [DOI: 10.1183/23120541.00604-2021]**	The study was excluded because the population consists of non-purulent exudative pleural effusions rather than adults with complicated parapneumonic effusion, empyema, or pleural infection. Therefore, it does not match the target population.
**Lui 2021 [DOI: 10.1016/j.rmed.2021.106553]**	The study was excluded because it is a review/management article on pleural sepsis and does not provide original extractable data on routinely available prognostic factors and eligible clinical outcomes.
**McGuire 2020 [DOI: 10.1080/24745332.2019.1598830]**	The study was excluded because it evaluates predictors of progression from pneumonia to empyema or complex parapneumonic effusion. The review question focuses on prognostic factors after complicated parapneumonic effusion or empyema has already developed.
**Karpickij 2024 [DOI: 10.33029/2308-1198-2024-12-3-121-127]**	The study was excluded because it concerns pleural empyema associated with COVID-19 pneumonia and does not clearly report an eligible routinely available prognostic factor-outcome association for treatment failure or mortality.
**Yayan 2026 [PMID: 41952233]**	The study was excluded because it compares VATS with thoracotomy in patients treated surgically for empyema. It is primarily a therapeutic comparison and does not focus on routinely available prognostic factors associated with treatment failure or mortality.
**Oh 2025 [PMID: 41267088]**	The study was excluded because the main predictor is thoracic sarcopenia measured on computed tomography. This is an advanced imaging-derived marker rather than a routinely available clinical, biochemical, microbiological, or pleural fluid marker within the scope of the review.
**Suwanarawat 2025 [PMID: 41066326]**	The study was excluded because it compares VATS with open thoracotomy for organized empyema. The study is primarily therapeutic/surgical and does not provide an eligible analysis of routinely available prognostic factors.
**Lin 2024 [PMID: 39427034]**	The study was excluded because it primarily evaluates the optimal timing of VATS decortication for chronic empyema. The focus is surgical timing rather than routinely available prognostic factors for treatment failure or mortality.
**Galata 2024 [PMID: 38597111]**	The study was excluded because the main predictor is thoracic skeletal muscle mass measured on computed tomography. This is an imaging-derived specialised marker and falls outside the planned scope of routinely available clinical, biochemical, microbiological, or pleural fluid factors.
**Semmelmann 2023 [PMID: 37236839]**	The study was excluded because it focuses on postoperative pulmonary complications and perioperative/anesthetic factors after surgery for pleural empyema, rather than routinely available pre-treatment prognostic factors for

	treatment failure or mortality.
**Báez-Saldaña 2021 [PMID: 33498075]**	The study was excluded because it is mainly descriptive, reporting clinical and microbiological characteristics and treatment patterns in parapneumonic effusion or empyema without a clear extractable predictor-outcome association.
**Nayak 2019 [PMID: 31356799]**	The study was excluded because it is a population-based comparison of operative versus nonoperative treatment for thoracic empyema. It is primarily therapeutic and does not focus on routinely available prognostic factors.
**Samancilar 2018 [PMID: 29343663]**	The study was excluded because it compares VATS and intrapleural fibrinolysis. This is a therapeutic comparison without a clear eligible analysis of routinely available prognostic factors associated with adverse outcomes.
**Kondov 2017 [PMID: 28991771]**	The study was excluded because it reports surgical treatment results in pleural empyema but does not clearly provide an eligible predictor-outcome analysis using routinely available prognostic factors.
**Bongiolatti 2017 [PMID: 28108575]**	The study was excluded because it focuses on uniportal VATS and ultrasonographic staging. The emphasis is surgical/technical and imaging-based rather than routinely available prognostic factors for treatment failure or mortality.
**Li 2016 [PMID: 27276396]**	The study was excluded because it evaluates pleural adenosine deaminase as a diagnostic/microbiological biomarker to distinguish Gram-negative from Gram-positive pleural space infections. It does not assess treatment failure, mortality, or another eligible adverse prognostic outcome.

Table S2. Quality Assessment of Included Articles using the Newcastle-Ottawa Scale.

STUDY	SELECTION/4	COMPARABILITY/2	OUTCOME/3	TOTAL/9	RISK OF BIAS
**Wong 2025 [27]**	★★★★	★★	★★★	9	**Low**
**Liu 2025 — respiratory cultures [28]**	★★★	★	★★★	7	**Moderate**
**Yong 2025 [29]**	★★★★	★★	★★	8	**Low**
**Kim 2024 [19]**	★★★★	★★	★★★	9	**Low**
**Misthos 2005 [15]**	★★★★	★★	★★	8	**Low**
**Simon 2023 [20]**	★★★★	☆☆	★★★	7	**Moderate**
**Kim 2014 [16]**	★★★★	★★	★★★	9	**Low**
**Davies 1999 [14]**	★★★★	☆☆	★★	6	**Moderate**
**Akboga 2026 [21]**	★★★	☆☆	★★	5	**High**
**Chen 2014 [17]**	★★★★	★★	★★★	9	**Low**
**Mwesige 2025 [22]**	★★★★	★★	★★★	9	**Low**
**Shiroshita 2025 [23]**	★★★★	★★	★★★	9	**Low**
**Sedhai 2025 [24]**	★★★	☆☆	★★★	6	**Moderate**
**Meyer 2018 [35]**	★★★★	★★	★★★	9	**Low**
**Touray 2018 [36]**	★★★	★	★★★	7	**Moderate**
**Ahn 2018 [37]**	★★★	★	★★★	7	**Moderate**
**Wu 2018 [38]**	★★★★	★★	★★★	9	**Low**
**Tantraworasin 2018 [39]**	★★★★	★★	★★	8	**Low**
**Khemasuwan 2025 [30]**	★★★★	★★	★★	8	**Low**
**Carneiro 2024 [31]**	★★★	★	★★★	7	**Moderate**

**Stüben 2023 [32]**	★★★★	★★	★★	8	**Low**
**Corcoran 2020 [33]**	★★★★	★★	★★★	9	**Low**
**Tsai 2019 [34]**	★★★★	★★	★★	8	**Low**
**Liu 2025 — recurrence [25]**	★★★★	★★	★★	8	**Low**
**Hansen 2025 [26]**	★★★★	★★	★★★	9	**Low**
**Park 2016 [40]**	★★★★	★★	★★★	9	**Low**
**Koma 2017 [41]**	★★★★	☆☆	★★★	7	**Moderate**
**Kim 2016 [43]**	★★★★	★★	★★★	9	**Low**
**Schweigert 2016 [42]**	★★★★	★★	★★★	9	**Low**
**Salim 2016 [43]**	★★★★	★★	★★★	9	**Low**

*Scoring: The Newcastle–Ottawa Scale (NOS) was used to assess the methodological quality of the included cohort studies. The scale evaluates three domains: Selection (maximum 4 points), Comparability (maximum 2 points), and Outcome (maximum 3 points), yielding a maximum score of 9 points. Studies scoring 8–9 points were considered to have a low risk of bias (high methodological quality), those scoring 6–7 points were considered to have a moderate risk of bias (moderate methodological quality), and those scoring ≤5 points were considered to have a high risk of bias (low methodological quality).

Table S3. Study-Level Data Used in the Meta-Analysis of RAPID Score and Short-Term Mortality.

Study	Reference	Follow-up	High-risk deaths / total	Low/intermediate-risk deaths / total	Individual OR	95% CI	Random-effects weight (%)
Corcoran 2020 (PILOT)	[33]	3 months	27 / 92	26 / 450	6.77	3.72–12.32	54.6
Touray 2018	[36]	90 days	7 / 31	5 / 67	3.62	1.05–12.51	12.7
Hansen 2024	[26]	3 months	13 / 33	9 / 89	5.78	2.17–15.41	20.3
Carneiro 2024	[31]	3 months	4 / 10	1 / 24	15.33	1.44–163.76	3.5
Akboga 2026	[21]	In-hospital / within 90 days	6 / 26	3 / 55	5.20	1.19–22.82	8.9
**Pooled estimate**	—	**3 months / 90 days**	**57 / 192**	**44 / 685**	**6.09**	**3.25–11.38**	**100.0**

Table S4. Study-Level Data Used in the Meta-Analysis of Age and Short-Term Mortality.

**Study**	**Sample size**	**Mortality time point**	**Adjusted OR per 1 year (95% CI)**	**Converted OR per 10 years (95% CI)**	**Random-effects weight**
**Chen 2014 [17]**	602	In-hospital mortality	1.031 (1.008-1.054)	1.36 (1.08-1.69)	39.7%
**Meyer 2018 [35]**	437	90-day mortality	1.060 (1.040-1.090)	1.79 (1.48-2.37)	37.4%
**Mwesige 2025 [22]**	200	In-hospital mortality	1.040 (1.010-1.080)	1.48 (1.10-2.16)	23.0%
**Pooled estimate**	**1,239**	**Short-term mortality**	**—**	**1.54 (1.02-2.30)**	**100%**

*Random-effects model using the restricted maximum likelihood estimator and modified Hartung-Knapp confidence interval. Pooled OR 1.54 (95% CI 1.02-2.30); $p=0.045$; $I^2=30.4\%$; $tau^2=0.009$; $Q=2.87$, $p=0.238$. Studies reporting age using non-comparable categorical thresholds, unadjusted group summaries, or internally inconsistent effect directions were not pooled.
